# Topical Spironolactone in the Treatment of Evaporative Dry Eye Disease

**DOI:** 10.7759/cureus.41038

**Published:** 2023-06-27

**Authors:** Calvin W Wong, Brian S Wong, Wajahat Ali, Mikhail L De Jesus, Tatiana A Melber, Richard W Yee

**Affiliations:** 1 Ophthalmology, University of Texas Health Science Center at Houston, Houston, USA; 2 Department of Radiology, University of Texas Medical Branch at Galveston, Galveston, USA; 3 Department of Psychiatry, University of Texas Medical Branch at Galveston, Galveston, USA; 4 Medicine, University of Connecticut Health, Farmington, USA; 5 Public Health, Montgomery County Department of Public Health, Conroe, USA; 6 Head and Neck Surgery, MD Anderson Cancer Center, Houston, USA

**Keywords:** cornea and external eye diseases, ocular surface disease, dry eye syndrome, evaporative dry eye, dry eye disorder

## Abstract

Meibomian gland dysfunction (MGD) is associated with evaporative dry eye syndrome, which is characterized by a reduction in meibum secretion and tear film instability. Present treatments provide only temporary relief, thereby necessitating the exploration of novel therapeutic strategies for chronic treatment. This study aims to evaluate topical spironolactone, a medication with anti-mineralocorticoid, anti-androgenic, and anti-inflammatory properties, in treating dry eye. A retrospective observational study was performed on the medical records of 102 patients diagnosed with dry eye disease. These patients were categorized into two groups based on their Schirmer's tear test scores. Various clinical indicators, including subjective global assessment scores, visual acuity, keratitis, conjunctival staining scores, and lid margin health, were evaluated prior to and following treatment with topical spironolactone eye drops. The group with higher Schirmer's scores exhibited improvement in self-reported global assessment scores after treatment. Significant improvements were also observed in keratitis and conjunctival staining scores, visual acuity, and lid margin inflammation. Similarly, the group with lower Schirmer's scores demonstrated improvements in self-reported global assessment scores and visual acuity after treatment. Topical spironolactone may improve tear film quality and address the inflammatory processes associated with MGD and evaporative dry eye. Moreover, the topical administration of spironolactone in an ocular vehicle appears to be well tolerated and may mitigate the risk of systemic adverse effects. Further studies are warranted to explore the long-term effects of topical spironolactone in the treatment of evaporative dry eye disease.

## Introduction

Meibomian gland dysfunction (MGD) is a common contributor to dry eye syndrome, which results from a reduced secretion of oily substances by meibomian glands that help maintain tear film stability. The glands of affected individuals often show terminal duct obstruction, inflammation, and changes in glandular secretion. The condition is diagnosed based on various factors, including glandular dropout, reduced secretion upon gland expression, meibum secretion quality, inflammation, and meibography [1,2]. Treatments for MGD in the setting of evaporative dry eye include warm compresses, lid hygiene, intraductal meibomian gland probing, lipid-emulsion eye drops, thermal pulsation, n-acetyl-cysteine, azithromycin, omega-3 fatty acid supplementation, cyclosporine A eye drops, and intense pulse-light therapy [3,4].

Spironolactone is a drug commonly used to treat heart failure that has anti-mineralocorticoid and anti-androgenic effects [5]. Off-label uses include the treatment of hirsutism, female-pattern hair loss, and hormonal acne [5,6]. In addition to its classical anti-mineralocorticoid and anti-androgenic activity, spironolactone has been shown to upregulate PPAR-gamma and TGF-β [7,8], which results in an anti-fibrotic and anti-inflammatory effect [7]. Mineralocorticoid receptor signaling has been implicated in oxidative stress and vascular pathologies. A growing corpus of work documents an increasing number of diverse signaling pathways and novel mechanisms associated with mineralocorticoid receptor activation and blockade [9-12]. Other work has shown inhibition of macrophage recruitment in corneal tissue, inhibition of corneal neovascularization, and improved corneal re-epithelialization after injury with the application of spironolactone in a mouse model [13]. Last, in vitro and in vivo topical application of spironolactone has shown an association with increased lipid density and presence in the corneal epithelium [14,15]. Thus, topical spironolactone may improve the quality of the tear film and address inflammation in patients with MGD and evaporative dry eye.

While spironolactone has been associated with adverse effects such as increased urinary frequency, hyperkalemia, rashes, gynecomastia in men, and menstrual irregularities in women, its long-term use appears to be safe [16,17]. The use of topical spironolactone in an ocular vehicle has not been previously reported, but it may reduce the risk of undesirable or adverse effects because of decreased systemic levels compared to oral therapy.

This study aims to evaluate the efficacy of topical spironolactone in the treatment of ocular surface disease based on subjective global assessment scores, visual acuity, keratitis, and conjunctival staining scores. Recognizing the importance of lid margin health and anatomy in evaporative dry eye, anterior blepharitis grade, lid margin vascularity grade, obstruction grade, turbidity grade, and zone A grade were assessed [18,19]. Additionally, the study aims to evaluate whether the efficacy of topical spironolactone differs in patients with aqueous tear deficiency compared to patients who make adequate tears, as seen in Schirmer's tear scores.

Current treatments for evaporative dry eye disease offer temporary relief for an otherwise chronic disease. Spironolactone, a drug with anti-androgenic, anti-mineralocorticoid, and anti-inflammatory properties, may improve the quality of the tear film and address fibrotic or inflammatory processes in patients with MGD. The study aims to evaluate the efficacy of topical spironolactone in the treatment of ocular surface disease.

## Materials and methods

The study aimed to retrospectively evaluate the efficacy of off-label use of spironolactone (0.3%) eye drops in treating patients with dry eye disease as measured by a variety of clinical indicators in a pre- and post-treatment comparison. The medical records of 102 patients managed at an urban dry eye-based practice were reviewed. Patients were selected based on the quality of documentation and inclusion criteria involving a diagnosis of dry eye disease by aqueous deficiency, conjunctivitis, or a patient complaint of dryness. Patients that met inclusion criteria were divided into two groups based on their Schirmer’s test scores: the first group included 75 patients with scores greater than 5 mm, and the second group included 27 patients with scores of 5 mm or less. This study was conducted in accordance with the Declaration of Helsinki and under institutional review board criteria.

Two time points were selected for statistical analysis: the visit at which patients started using topical spironolactone and the subsequent follow-up visit. The subjective global assessment score was collected based on patients' self-assessment of their dry eye symptoms based on a standardized verbal questionnaire, with severity scores ranging from 0 to 10. The severity of keratitis and conjunctival findings (graded 0 to 3) was determined by lissamine green staining of the nasal conjunctival, central corneal, and temporal conjunctival areas. Unanesthetized Schirmer's tests were done to evaluate tear film production, and visual acuity was recorded at both visits. All questionnaires and clinical findings were collected by the same individual.

A slit lamp examination was used to assess the severity of anterior blepharitis, while abnormalities in the lid margin, such as vascularity and inflammation in the avascular region (zone A), were graded on a scale ranging from 0 to 4. Meibomian gland obstruction was evaluated by applying pressure to the lower lid and graded on a scale from 0 to 4, and the quality of the meibum that was expressed was also graded on a scale from 0 to 4. The diagnosis of MGD was established if the symptoms scored 3+ or the lid margin abnormalities scored 2+. Exclusion criteria included a history of potentially confounding diseases such as Sjogren's syndrome, ocular rosacea, and pemphigoid, as well as the use of glaucoma medications or steroid eye drops.

Prior to starting spironolactone eye drops, patients had not tried other pharmacological treatments for dry eye. However, some patients had already tried more conservative treatments like omega-3 fatty acid and flax seed oil supplements with limited improvement. The spironolactone eye drops were dosed twice daily in both eyes until their first follow-up visit, which was on average one month after the first visit.

Statistical analysis was performed using STATA 13 (StataCorp LLC, Texas, USA), and non-parametric and paired t-tests were used to analyze the data. Overall, the study aimed to investigate the efficacy of spironolactone eye drops in treating patients with dry eye disease and to evaluate the impact of treatment on various clinical indicators.

## Results

Evaporative dry eye

For the cohort of 75 patients with Schirmer’s scores greater than 5, the mean subjective global assessment score (0-10) prior to treatment was 5.32 ± 2.12 (Table 1).

**Table 1:** Pre-treatment descriptive statistics for normal Schirmer’s cohort (n=75) OD: right eye; OS: left eye

Parameter	Mean	Standard deviation
Subjective global assessment	5.32	2.12
Keratitis OD temporal	0.49	0.64
Keratitis OD corneal	0.03	0.23
Keratitis OD nasal	0.77	0.71
Keratitis OS nasal	0.84	0.77
Keratitis OS corneal	0.07	0.29
Keratitis OS temporal	0.64	0.63
Anterior blepharitis	0.35	0.82
Vascularity	1.42	0.90
Obstruction	1.35	0.81
Turbidity	2.68	0.54
Zone A	3.07	0.80
Vision OD (logMAR units)	0.09	0.17
Vision OS (logMAR units)	0.13	0.23
Schirmer’s OD (mm)	16.31	8.28
Schirmer's OS (mm)	15.97	7.77

The same cohort after treatment with spironolactone showed a reduction in the mean subjective global assessment score to 4.03 ± 1.57 (Table 2).

**Table 2:** Post-treatment descriptive statistics: normal Schirmer’s (n=75) OD: right eye; OS: left eye

Parameter	Mean	Standard deviation
Subjective global assessment	4.03	1.57
Keratitis OD temporal	0.45	0.64
Keratitis OD corneal	0.01	0.06
Keratitis OD nasal	0.64	0.68
Keratitis OS nasal	0.72	0.73
Keratitis OS corneal	0.07	0.34
Keratitis OS temporal	0.57	0.69
Anterior blepharitis	0.39	0.67
Vascularity	1.11	0.83
Obstruction	0.98	0.69
Turbidity	1.75	0.75
Zone A	2.51	0.88
Vision OD (logMAR units)	0.08	0.18
Vision OS (logMAR units)	0.11	0.22
Schirmer’s OD (mm)	16.47	9.75
Schirmer's OS (mm)	16.95	9.61

Self-reported global assessment scores showed a statistically significant improvement of 1.29 ± 1.80 (p=0.00) units after starting topical spironolactone (Table 3).

**Table 3:** Pre-treatment vs post-treatment statistics: normal Schirmer’s (n=75) OD: right eye; OS: left eye

Parameter	Mean difference	Standard deviation	p-value
Subjective global assessment	−1.29	1.80	<0.01
Keratitis OD temporal	−0.03	0.65	0.99
Keratitis OD corneal	−0.02	0.24	0.99
Keratitis OD nasal	−0.13	0.56	0.22
Keratitis OS nasal	−0.12	0.67	0.24
Keratitis OS corneal	0.00	0.32	0.43
Keratitis OS temporal	−0.06	0.59	0.43
Anterior blepharitis	0.04	0.70	0.65
Vascularity	−0.31	0.71	<0.01
Obstruction	−0.36	0.90	<0.01
Turbidity	−0.93	0.73	<0.01
Zone A	−0.55	0.83	<0.01
Vision OD (logMAR units)	−0.02	0.08	0.06
Vision OS (logMAR units)	−0.02	0.10	0.25
Schirmer’s OD (mm)	0.11	8.07	0.45
Schirmer's OS (mm)	0.92	9.44	0.20

Prior to treatment, mean right eye keratitis and conjunctival staining scores were 0.49 ± 0.64, 0.03 ± 0.23, and 0.77 ± 0.71 in the temporal, corneal, and nasal regions, respectively. In the left eye, the pretreatment mean keratitis and conjunctival staining scores were 0.64 ± 0.63 (temporal), 0.07 ± 0.29 (central), and 0.84 ± 0.77 (nasal). At the first follow-up, keratitis and conjunctival score means were 0.45 ± 0.64, 0.007 ± 0.06, and 0.64 ± 0.68 in the temporal, corneal, and nasal regions of the right eye and 0.57 ± 0.69, 0.07 ± 0.34, and 0.72 ± 0.73 in the left eye, respectively.

Overall, the mean change in keratitis and conjunctival scores was −0.03 ± 0.65 (p=0.99), −0.02 ± 0.24 (p=0.99), and −0.13 ± 0.56 (p=0.22) in the temporal, corneal, and nasal regions of the right eye, and −0.06 ± 0.59 (p=0.43), 0 ± 0.32 (p=0.43), and −0.12 ± 0.67 (p=0.24) in the left eye (Table 3).

**Figure 1:**
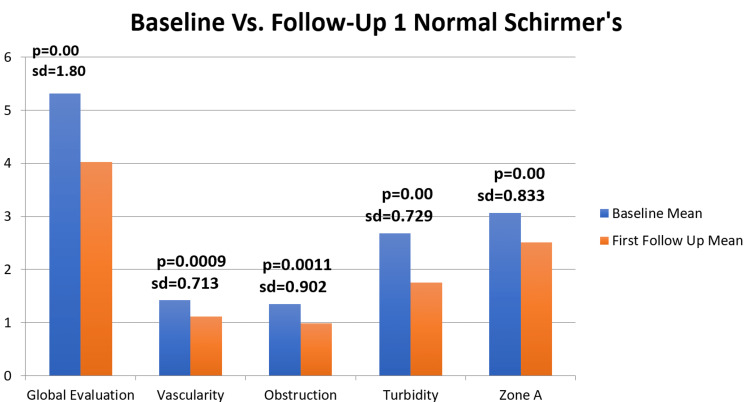
Significant statistical measurements for baseline versus follow up - normal Schirmer’s (n=75)

Statistically significant differences from pre- and post-treatment metrics suggested reductions in mean subjective global assessment, vascularity, obstruction, turbidity, and Zone A scores (Figure 1).

The mean logarithm mean adjusted ratio (logMAR) score of patients pre-treatment was 0.09 ± 0.17 and 0.13 ± 0.23 logMAR units in the right and left eyes, respectively (Table 1). Post-treatment, the logMAR score was 0.08 ± 0.18 and 0.11 ± 0.22 units in the right and left eyes (Table 2). The mean change in logMAR units was −0.016 ± 0.08 units (p = 0.0562) for the right eye and −0.018 ± 0.095 log units (p = 0.248) for the left.

The mean anterior blepharitis score was 0.35 ± 0.82 prior to treatment and 0.39 ± 0.67 post-treatment; the mean change in anterior blepharitis score was 0.04 ± 0.70 (p = 0.6475). The mean vascularity score was 1.42 ± 0.897 prior to treatment and 1.11 ± 0.829 post-treatment, with a mean improvement of 0.31 ± 0.713 (p = 0.0009). The mean obstruction score was 1.35 ± 0.81 prior to treatment and 0.98 ± 0.69 post-treatment, with a mean improvement of 0.36 ± 0.90 (p = 0.0011). The mean turbidity score was 2.68 ± 0.543 prior to treatment and 1.75 ± 0.75 post-treatment, with a mean improvement of 0.93 ± 0.73 (p <0.01). The mean zone A score was 3.07 ± 0.80 prior to treatment and 2.51 ± 0.88 post-treatment, with a mean improvement of 0.53 ± 0.83 (p <0.01). The Schirmer’s score prior to treatment was 16.31 ± 8.28 and 15.97 ± 7.78 for the right and left eyes, respectively. Post-treatment, the Schirmer’s scores were 16.47 ± 9.75 and 16.95 ± 9.61, with a mean improvement of 0.11 ± 8.07 (p = 0.454) and 0.92 ± 9.43 (p = 0.204) for the right and left eyes, respectively.

Aqueous-deficient dry eye

The cohort of 27 patients with aqueous-deficient dry eyes was defined by Schirmer’s scores of less than 5. For this cohort, the mean pre-treatment subjective global assessment score of the MGD was 4.76 ± 2.37 (Table 4).

**Table 4:** Pre-treatment descriptive statistics for low Schirmer’s cohort (n=27) OD: right eye; OS: left eye

Parameter	Mean	Standard deviation
Subjective global assessment	4.76	2.37
Keratitis OD temporal	0.94	1.06
Keratitis OD corneal	0.20	0.52
Keratitis OD nasal	0.98	0.87
Keratitis OS nasal	1.04	0.88
Keratitis OS corneal	0.15	0.37
Keratitis OS temporal	0.92	0.93
Anterior blepharitis	0.41	0.83
Vascularity	1.26	0.90
Obstruction	1.52	0.88
Turbidity	2.57	0.51
Zone A	3.09	0.92
Vision OD (logMAR units)	0.08	0.14
Vision OS (logMAR units)	0.15	0.28
Schirmer’s OD (mm)	4.91	4.05
Schirmer's OS (mm)	4.60	3.73

The post-treatment mean subjective global assessment score was 3.78 ± 1.68 (Table 5).

**Table 5:** Post-treatment descriptive statistics: low Schirmer’s (n=27) OD: right eye; OS: left eye

Parameter	Mean	Standard deviation
Subjective global assessment	3.78	1.68
Keratitis OD temporal	0.74	0.91
Keratitis OD corneal	0.07	0.38
Keratitis OD nasal	0.93	0.84
Keratitis OS nasal	1.19	0.87
Keratitis OS corneal	0.15	0.37
Keratitis OS temporal	0.96	0.82
Anterior blepharitis	0.41	0.71
Vascularity	0.93	0.83
Obstruction	1.13	1.05
Turbidity	1.89	0.91
Zone A	2.46	1.13
Vision OD (logMAR units)	0.05	0.12
Vision OS (logMAR units)	0.13	0.30
Schirmer’s OD (mm)	7.52	7.42
Schirmer's OS (mm)	6.92	5.34

The patients had an improvement of 0.98 ± 2.18 (p=0.0297) in self-reported global assessment scores (Table 6). 

**Table 6:** Pre-treatment versus post-treatment statistics: low Schirmer’s (n=27) OD: right eye; OS: left eye

	Mean difference	Standard deviation	p-value
Subjective global assessment	−0.98	2.18	0.03
Keratitis OD temporal	−0.20	0.75	0.35
Keratitis OD corneal	−0.13	0.55	0.30
Keratitis OD nasal	−0.06	0.53	0.40
Keratitis OS nasal	0.15	0.70	0.39
Keratitis OS corneal	0.00	0.40	1.00
Keratitis OS temporal	0.04	0.56	0.74
Anterior blepharitis	0.00	0.64	0.75
Vascularity	−0.33	0.55	0.01
Obstruction	−0.39	1.48	0.15
Turbidity	−0.69	0.76	0.00
Zone A	−0.63	1.21	0.02
Vision OD (logMAR units)	−0.03	0.08	0.03
Vision OS (logMAR units)	−0.02	0.08	0.21
Schirmer’s OD (mm)	2.61	7.03	0.03
Schirmer's OS (mm)	2.33	4.74	0.01

**Figure 2:**
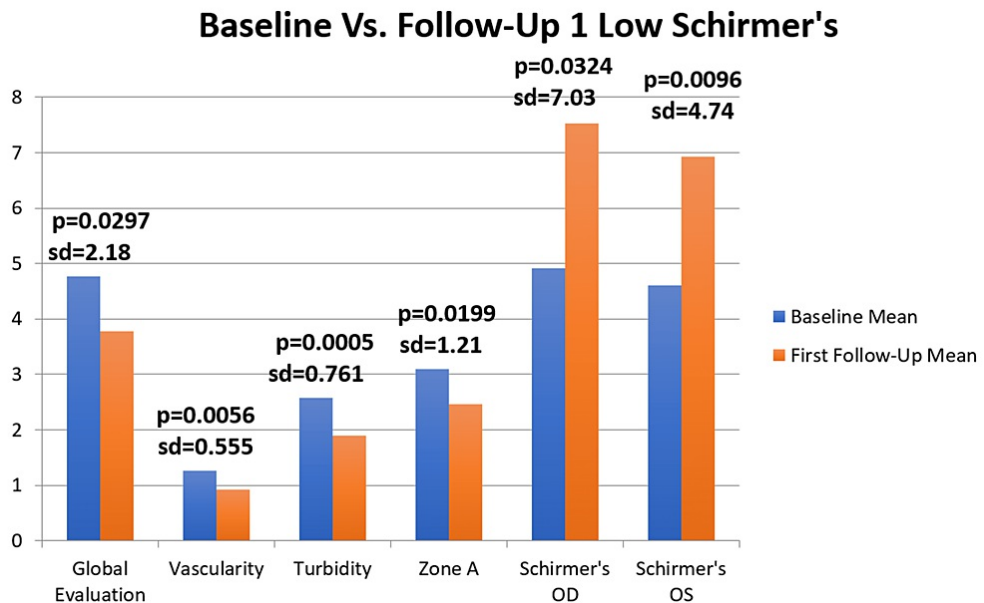
Significant statistical measurements for baseline versus follow-up 1 - low Schirmer’s (n=27) OD: right eye; OS: left eye

Before treatment, the average keratitis and conjunctival staining scores for the right eye were 0.94 ± 1.06, 0.20 ± 0.52, and 0.98 ± 0.87 in the temporal, corneal, and nasal regions, respectively. Similarly, for the left eye, the scores were 0.92 ± 0.93, 0.15 ± 0.37, and 1.04 ± 0.88 in the same regions. After treatment, the mean keratitis and conjunctival staining scores were 0.74 ± 0.91, 0.07 ± 0.38, and 0.93 ± 0.84 in the temporal, corneal, and nasal regions of the right eye, and 0.96 ± 0.82, 0.15 ± 0.37, and 1.19 ± 0.87 in the same regions of the left eye. The average changes in keratitis and conjunctival scores were −0.20 ± 0.75 (p=0.349), −0.13 ± 0.55 (p=0.2991), and −0.056 ± 0.53 (p=0.403) in the temporal, corneal, and nasal regions of the right eye, and 0.038 ± 0.56 (p=0.743), 0 ± 0.4 (p=1), and 0.15 ± 0.70 (p=0.386) in the same regions of the left eye (Figure 2).

Before treatment, the average visual acuity of patients was 0.08 ± 0.14 and 0.15 ± 0.28 logMAR units in the right and left eyes, respectively. Following treatment, the visual acuity improved to 0.05 ± 0.12 and 0.13 ± 0.30 logMAR units in the right and left eyes, respectively. The mean change in visual acuity was -0.0345 ± 0.08 (p = 0.0324) logMAR units for the right eye and −0.0198 ± 0.078 (p = 0.2112) for the left eye.

The average score for anterior blepharitis was 0.41 ± 0.83 before treatment and remained the same at 0.41 ± 0.71 after treatment. The mean change in the anterior blepharitis score was 0.0 ± 0.64 (p = 0.7535), indicating no significant difference. The mean vascularity score before treatment was 1.26 ± 0.90, which improved to 0.93 ± 0.829 after treatment. The mean improvement in the vascularity score was 0.33 ± 0.55 (p = 0.0056), indicating a statistically significant improvement. Prior to treatment, the mean obstruction score was 1.52 ± 0.88, which decreased to 1.13 ± 1.05 post-treatment. The mean improvement in the obstruction score was 0.39 ± 1.48 (p = 0.1531), but this change was not statistically significant. The mean turbidity score was 2.57 ± 0.51 before treatment, and it decreased to 1.89 ± 0.91 after treatment. The mean improvement in the turbidity score was 0.69 ± 0.76 (p < 0.01), indicating a significant improvement. For the zone A score, the mean score before treatment was 3.09 ± 0.92, which decreased to 2.46 ± 1.13 after treatment. The mean improvement in the zone A score was 0.63 ± 1.21 (p = 0.0199), showing a statistically significant improvement. The Schirmer's score prior to treatment was 4.91 ± 4.05 and 4.60 ± 3.73 for the right and left eyes, respectively. After treatment, Schirmer's scores increased to 7.52 ± 7.42 and 6.92 ± 5.34 for the right and left eyes, respectively. The mean improvement in Schirmer's score was 2.61 ± 7.03 (p = 0.0324) for the right eye and 2.33 ± 4.74 (p = 0.0096) for the left eye, indicating a significant improvement in both cases.

## Discussion

To the best of our knowledge, this study is the first to report on the use of topical spironolactone for dry eye disease, and the finding of improved clinical features such as lid margin vascularity, meibomian gland obstruction, and meibum turbidity may provide a rationale for the observed improvement in the subjective global assessment. Previous work on the use of topical anti-mineralocorticoids has shown improvements in ocular graft-versus-host Disease severity as well as lid margin vascularity, and the results of this study are consistent with such findings. The literature well documents the anti-fibrotic, anti-inflammatory, and pro-healing effects of anti-mineralocorticoids on various tissues like renal parenchyma, myocardium, choroid, and corneal epithelium [5,9-13,20]. Analogous effects on the ocular surface and adnexae may explain the improvement in lid margin parameters and subjective global comfort level across both aqueous-deficient and aqueous-sufficient groups.

Using topical spironolactone instead of oral administration offers the advantage of requiring a lower concentration to achieve the desired therapeutic effect directly at the targeted location. Nonetheless, topical application of spironolactone may still potentially result in adverse effects. A small proportion of patients experienced a mild and temporary burning sensation in the eye shortly after applying spironolactone, typically lasting no more than 30 seconds. Notably, no abnormal rises in intraocular pressure or abnormal vision changes were observed in association with topical spironolactone use.

The role of mineralocorticoid receptor blockade on the ocular surface and adnexae remains poorly elucidated, and possible mechanisms include the promotion of re-epithelialization after injury, inhibition of tissue fibrosis, and inhibition of inflammation and macrophage recruitment [13,20]. Additionally, the use of mineralocorticoid analogs to treat the inflammatory and evaporative etiologies of dry eye disease and MGD avoids many undesirable side effects associated with glucocorticoids. The safety profile of spironolactone appears suited for long-term, chronic management of dry eye disease.

Limitations of this study include the retrospective nature of this pilot study, the lack of a control group, and the small sample size. Further, the cohort with normal Schirmer’s (n=75) was larger than the cohort with low Schirmer’s (n=27). Based on the findings from this study, future work should focus on the effects of spironolactone on tear film physiology, and prospective masked placebo-controlled studies on the use of spironolactone for dry eye disease are warranted.

## Conclusions

The use of topical spironolactone for evaporative dry eye is a novel application of a drug used for decades to treat heart failure. Classical mineralocorticoid antagonism does not fully account for the growing corpus of literature documenting the antifibrotic, antiphagocytic, and lipogenic effects of spironolactone. While these effects have been well documented in the context of renal and cardiac remodeling in heart failure, mineralocorticoid receptor antagonism may address cicatricial, autoimmune, or evaporative facets of dry eye. This work documented improvements in subjective comfort level, lid margin inflammatory markers, and meibomian gland function after the use of topical spironolactone for four weeks on average. Mineralocorticoid receptor antagonism may provide a novel treatment option for complex dry eye patients with not only aqueous deficiency but also inflammatory and evaporative etiologies of dry eye.
